# Autophagy Modulators: Mechanistic Aspects and Drug Delivery Systems

**DOI:** 10.3390/biom9100530

**Published:** 2019-09-25

**Authors:** Shima Tavakol, Milad Ashrafizadeh, Shuo Deng, Maryam Azarian, Asghar Abdoli, Mahsa Motavaf, Delaram Poormoghadam, Hashem Khanbabaei, Elham Ghasemipour Afshar, Ali Mandegary, Abbas Pardakhty, Celestial T. Yap, Reza Mohammadinejad, Alan Prem Kumar

**Affiliations:** 1 Cellular and Molecular Research Center, Iran University of Medical Sciences, Tehran P.O. Box 1449614525, Iran; shima.tavakol@yahoo.com; 2 Department of basic science, Faculty of Veterinary Medicine, University of Tabriz, Tabriz 1455742, Iran; dvm.milad73@yahoo.com; 3 Department of Physiology, Yong Loo Lin School of Medicine, National University of Singapore, Singapore 117597, Singapore; phsdes@nus.edu.sg (S.D.); phsyapc@nus.edu.sg (C.T.Y.); 4 Department of Biology, Science and Research Branch, Islamic Azad University, Tehran 1477893855, Iran; azarian.maryam@gmail.com; 5 Departament de Bioquímica i Biologia Molecular, Institut de Biotecnologia i Biomedicina (IBB), Universitat Autónoma de Barcelona, 08193 Barcelona, Spain; 6 Department of Hepatitis and AIDS, Pasteur Institute of Iran, Tehran 1316943551, Iran; asghar.abdoli7@gmail.com; 7 Department of Molecular Genetics, Faculty of Biological Sciences, Tarbiat Modares University, Tehran 14115-111, Iran; motavaf.m@gmail.com; 8 Department of Medical Nanotechnology, Faculty of Advanced Sciences & Technology, Pharmaceutical Sciences Branch, Islamic Azad University, (IAUPS), Tehran P.O. Box 1916893813, Iran; parand.pdrm@yahoo.com; 9 Medical Physics Department, School of Medicine, Ahvaz Jundishapur University of Medical Sciences, Ahvaz 61357-15794, Iran; khanbabaie.mph@gmail.com; 10 Neuroscience Research Center, Institute of Neuropharmacology, Kerman University of Medical Sciences, Kerman P.O. Box 7616911319, Iran; elham_gh_afshar@yahoo.com (E.G.A.); alimandegary@yahoo.com (A.M.); drpardakhti@yahoo.com (A.P.); 11 Pharmaceutics Research Center, Institute of Neuropharmacology, Kerman University of Medical Sciences, Kerman P.O. Box 1355576169, Iran; 12 Department of Pharmacology, Yong Loo Lin School of Medicine, National University of Singapore, Singapore 117 600, Singapore; 13 Cancer Science Institute of Singapore, National University of Singapore, Singapore 1192288, Singapore

**Keywords:** autophagy, mTOR, AMPK, Nanocarriers, combination therapy, cancer, siRNA

## Abstract

Autophagy modulation is considered to be a promising programmed cell death mechanism to prevent and cure a great number of disorders and diseases. The crucial step in designing an effective therapeutic approach is to understand the correct and accurate causes of diseases and to understand whether autophagy plays a cytoprotective or cytotoxic/cytostatic role in the progression and prevention of disease. This knowledge will help scientists find approaches to manipulate tumor and pathologic cells in order to enhance cellular sensitivity to therapeutics and treat them. Although some conventional therapeutics suffer from poor solubility, bioavailability and controlled release mechanisms, it appears that novel nanoplatforms overcome these obstacles and have led to the design of a theranostic-controlled drug release system with high solubility and active targeting and stimuli-responsive potentials. In this review, we discuss autophagy modulators-related signaling pathways and some of the drug delivery strategies that have been applied to the field of therapeutic application of autophagy modulators. Moreover, we describe how therapeutics will target various steps of the autophagic machinery. Furthermore, nano drug delivery platforms for autophagy targeting and co-delivery of autophagy modulators with chemotherapeutics/siRNA, are also discussed.

## 1. Introduction

Autophagy—cell self-digestion machinery—is a substantial process which plays critical roles in many cellular processes and functions. The most important type of autophagy is macroautophagy [1,2,3,4,5]. This process targets the damaged cytoplasmic proteins or organelles with different complexity and size [6,7,8]. In the autophagy, double-membrane structures (termed phagophore or isolation membrane) capture the cargo, including aged-proteins, injured-organelles, and pathogens, and then elongate into enclosed double-membraned autophagosomes [9,10,11]. Subsequently, autophagosomes are fused with lysosomes by merging the outer membrane of autophagosome with lysosomal membrane, resulting in the formation of autolysosomes [12,13,14]. Finally, the cargo being delivered, together with the inner autophagosome membrane, is broken down inside autolysosomes. This process, like a thrifty source in the cell, leads to the recycling of biomolecules during starvation. Therefore, autophagy is considered to be a homeostatic mechanism to conserve cell survival during stress conditions via the degradation of damaged cellular components and the recycling of cellular constituents [15,16,17,18].

Triggering of autophagy is induced through multiple intracellular and extracellular stimuli including infection, proton concentration, starvation, metabolic perturbations and other chemical and physical stressors. It is noteworthy that deregulated autophagy leads to several disorders not only in healthy situations but also in transformed mammalian cells [19]. Furthermore, autophagy exhibits cytoprotective impacts on cells that make them a vital player in the adaptive responses to intrinsic or extrinsic impulses. There are only a limited number of cases where autophagy has been known as the bona fide cause of regulated cell death [18]. Autophagy inhibition through diverse mechanisms for example drug or genetic, resulting in enhanced cell sensitivity to various stressors. In the importance of autophagy balance in cells, it should be said that not only permanent but also transient disturbance in autophagy leads to developmental and embryonic defects along with several pathological conditions.

As mentioned above, accurate protein homeostasis (proteostasis) and elimination of damaged or exacerbated intracellular compounds are crucial for the cell survival and its proper function. During starvation, autophagy is activated through multiple signaling pathways, such as mechanistic target of rapamycin (mTOR) pathway, one of the critical pathways involved in cell proliferation that inhibits autophagy [15,20]. Therefore, disturbances in autophagy, as the adverse effects of mTOR pathway, can create disorders. It is thought that autophagy has some protective and therapeutic roles in microbial infection, neurodegeneration, cardiovascular disorders and a dual role in cancer. Nevertheless, rapamycin promotes autophagy [21], and it seems that it may be prescribed as one of the choices in the treatment of the diseases which are caused by the inhibition of autophagy [22]. For example, in cancer cells, based on the different type and stage of cancer, nutrient availability, stress, immune system, and genetic context [23,24,25], autophagy will be induced and inhibited and this necessitates more investigations. There are several reports indicated that autophagy is inhibited at the onset of cancer in some parts through mTOR, Bcl2, damage-regulated autophagy modulator (DRAM) and PI3K activation and over-expression and P53 down-regulation. However, others believe that the level of cytoplasmic P53 not pool P53 leads to autophagy decrement at the basal level [7,26]. It is important to note that autophagy decrement at the just is sufficient for the onset of cancer and does not guarantee tumor progression [8]. However, the over-expression of P62 derived from autophagy inhibition leads to tumor progression through the increasing of ROS, NFκB, NRF2 and DNA damage [27]. Moreover, it seems that autophagy is elevated in advanced cancers. An important point is related to the modulatory effect of autophagy on immune system where elevated autophagy in advanced cancers increases high-mobility group box 1 protein (HMGB1) release [28]. These events result in inducing anti-tumor T-cell responses through the activation of Toll-like receptors. Therefore, inhibition of autophagy by chemotherapeutic agents will lead to a decrease in HMGB1 release and anti-tumor response.

Autophagy activation has a dual role in cancers. From one side, autophagy activation in cancer cells promotes the efficacy of anti-cancer strategies especially in the case of a functional immune system. From the other side, it may promote cancer progression through the enhancement of cell survival. In other hands, if the association of autophagy with multidrug resistance (MDR) is fortified [15], autophagy can be undoubtedly considered as a promising target in oncotherapy [29]. In other words, since the up-regulation of ABC transporters involved in MDR is correlated with the level of microtubule-associated protein 1A/1B-light chain 3 (LC3) and Beclin1, autophagy is in good agreement with MDR [30]. Besides, there are the relationships with the over-expression of LC3 and other biomolecules and miRNAs such as HMGB1and miR-199a-5p involved in MDR [31,32]. 

Importantly, inhibition of autophagy may conquer resistance to kinase inhibitors in cancer cells. Notably, the positive impact of autophagy on cancer therapy is dependent to the stage of cancer and its progression and if mTOR inhibitor pharmaceuticals failed in cancer therapy owing to the acidic pH microenvironment of cancer cells [33]. All together findings emphasize the importance of engineerable drug delivery systems to improve the efficacy of autophagy modulators.

Although these findings highlight the fact that targeting autophagy is of importance in the treatment of pathological conditions, particularly cancer, there are several drawbacks associated with currently applied autophagy modulators. It is held that autophagy modulators suffer from low bioavailability restricting their therapeutic efficiency. Additionally, non-targeted delivery is another pitfall associated with autophagy modulators. On the other hand, nanocarriers have demonstrated great potential in delivery of autophagy modulators [34]. To date, several nanocarriers such as liposomes [35,36], niosomes [37], micelles [38,39], carbon dots (CDs) [40] and polymeric ones [41,42] have been applied for delivery of drugs. In the present review, we describe the basics of autophagy with an emphasis on the molecular signaling pathways and demonstrate that how nanocarriers can aid in enhancing the efficacy of autophagy modulators.

## 2. mTOR Signaling Pathway

There are several anabolic and catabolic processes, which harmonize cell growth. Anabolism needs the energy to synthesize more sophisticated molecules from simple precursors such as fatty acids, amino acids, ATP, and nucleotides that are vital for cell growth and survival. In contrast, catabolism is a process, which releases energy and essential precursors to guarantee cell growth. Therefore, the balance of anabolic and catabolic processes is vital; however, the level of growth factors, nutrient and energy in cells, as well as hormonal inputs, define their balance. mTOR pathway plays a significant role in cell fate [43]. Importantly, mTOR protein kinase regulates some cellular anabolic processes and this has a remarkable role in autophagy inhibition [44,45,46,47]. This serine/threonine kinase pathway—which has been conserved from yeast to mammal [48]—consists of mTOR complex 1 (mTORC1) and mTORC2. These two sets are involved in the reception and coordination of different inputs such as growth signals, energy status and nutrients. Among them, mTORC1 has been deeply studied compared to the mTORC2 and has an important role in the enhancement of cell multiplication through regulating a variety of biosynthetic pathways. Besides, mTORC2 is considered to be the upstream of mTORC1 and is involved in cell morphology through the cytoskeletal organization. When the nutrients and energy are sufficient, the mTORC1 is activated and phosphorylates UlK1/2/FIP200/Atg13 complex [49,50], resulting in autophagy inhibition. After the inactivation of mTORC1, this complex becomes active via dephosphorylation and stimulates phagophore formation. The interesting point is related to the enabling autophagy at the basal level even if there are sufficient nutrients and energy in the cell in order to remove damaged organelles as well as aggregated or misfolded macromolecules. Due to their inhibitory role of autophagy initiation, mTORC inhbitors/activators have been extensively studied for their autophagy modulating effects and therapeutic application. 

Importantly, the mTOR inhibitor medications failed in clinical cancer therapy owing to the heterogeneity and mutation of mTOR [51]. The mTOR inhibitors are insensitive to the hypoxic region of tumors [52]. However, it appears the third generation of mTOR inhibitors, Rapalink, may be effective; they have a kinase inhibitor crosslinked with the rapamycin [53]. In the interim, Rapalog a conventional chemotherapeutic agent in cancer therapy is more effective in benign cancers and it seems that Rapalog is cytostatic rather than cytotoxic [54,55]. Nevertheless, Rapalog therapy suffers from significant side effects due to non-tissue and cell specificity, for example, metabolic, respiratory, dermatological, renal, and hematological toxicities [56]. In this way, for the combination therapy discussed in our study, rapamycin and Rapalog are not proposed. It is clear that the findings on the efficacy of ATP-competitive inhibitors of mTOR in clinical cancer therapy need more investigation [51,57].

A recently published article provides more details about the mTOR signaling pathway and its inhibitory effect on the autophagy process. It seems that mTORC1 has two distinct pools, known as endosome pool and vacuole pool. These pools play a significant role in mediating the modulatory impact of mTOR on autophagy. It appears that endosome pool has the major role in suppressing macroatuophagy and microautophagy by phosphorylation of Atg13 and Vps27, respectively, while vacuole pool contributes to enhancing the translation [58,59].

## 3. AMPK Signaling Pathway and Autophagy

**Figure 1:**
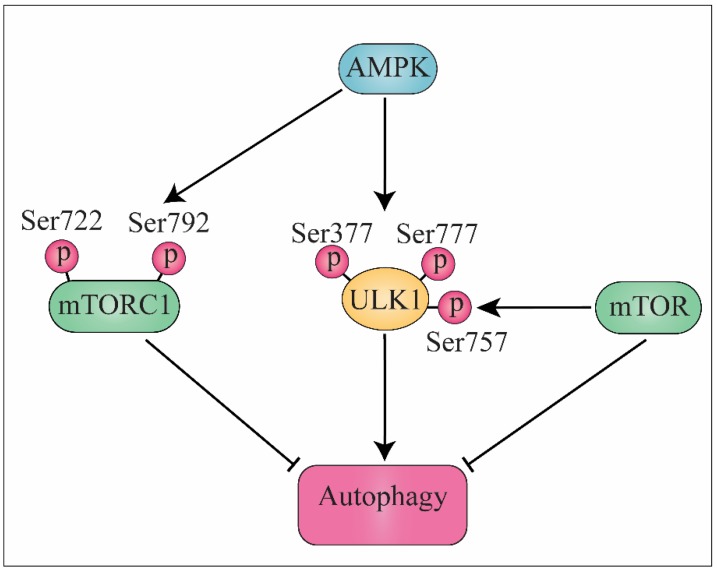
AMPK stimulates autophagy by phosphorylation of ULK1 at the Ser377 and Ser777. mTOR signaling pathway inhibits autophagy by phosphorylation of ULK1 at the Ser757. AMPK indirectly activates autophagy by suppressing mTORC1 via its phosphorylation at Ser722 and Ser792. AMPK, AMP-activated protein kinase; ULK1, unc-51 like autophagy activating kinase; mTOR, mechanistic target of rapamycin; mTORC1, mTOR complex 1.

Eukaryotes modulate their metabolism through adenosine monophosphate (AMP)-activated protein kinase (AMPK) signaling pathway as an evolved system based on their nutrient availability. AMPK is accounted as one of the crucial players in this system [60,61,62]. AMPK modulates four major pathways including protein, lipid and glucose metabolism, autophagy and mitochondrial homeostasis [63]. Notably, mTOR is also modulated through AMPK, inverse, mTOR modulates multiple direct or indirect target genes involved in the AMPK signaling pathway. In other words, under starvation, AMPK phoshprylates ULK1 (Ser317 and Ser777) to induce autophagy while under nutrient signal, mTOR phosphorylate ULK1 (Ser757) leads to disrupting AMPK- ULK1 interaction and autophagy inhibition [64]. However, APMK through the phosphorylation of TSC2 tumor suppressor (Ser1387) and raptor (Ser722 and Ser792) in mTORC1 directly inhibit mTOR (Figure 1) [65]. In summary, AMPK is involved as a guardian of metabolism and mitochondrial homeostasis. Maintaining homeostasis and equilibrium among anabolic and catabolic programs largely depends on AMPK signaling pathway and thereby it allows cells to provide an adequate response to metabolic stress [66]. Regarding AMPK performance in insulin signaling, glucose/lipid homeostasis, mitochondrial biogenesis and food intake, AMPK is recommended as one of the promising therapeutic objectives in the treatment of metabolic diseases such as obesity and type 2 diabetes [67].

Many studies have reported that AMPK gene over-expression has an important role in tumorigenesis [68]. An appropriate explanation could be the fact that LKB1 tumor suppressor acts as a key mediator upstream of AMPK kinase [69,70], even though, in the case of a mutation in the LKB1 gene, this can result in inherited Peutz–Jeghers syndrome, which could be due to the hamartomata’s polyps present in the intestine [71]. From another side, there are some controversial reports indicating AMPK may be effective in protecting the tumor cells versus limitation of nutrients, cytotoxic agents and hypoxia [72]. Therefore, there is a probability that the AMPK activators may harm the cancer treatment procedure [73].

Besides the association of AMPK with LKB1, AMPK influences mTORC1. mTORC1 and transcription initiation factor TIF-1A are involved in rapid cell proliferation and they are under AMPK control. In brief, upon AMPK activation, p21 up-regulation (cell cycle inhibitor protein), G1 phase cell cycle arrest and p53 activation are induced [74]. However, p53-AMPK-mTORC1 signaling pathways are under the regulation of p53-responsive gene products Sestrin1/2, as well [75]. To explain the above findings, it might be said that AMPK through engaging phosphorylation process inhibits cell cycle and in the meantime stabilizes cyclin-dependent kinase inhibitor p27kip1 as a reaction to stresses [76]. To sum things up and in agreement with the above mentioned studies, it seems that activation of AMPK signaling pathway using pharmaceuticals such as metformin, A-769662 and phenformin can defer the onset of tumorigenesis [77].

Another important feature of AMPK is its involvement in autophagy. Therefore, AMPK induces lysosomal-dependent catabolic program and leads to cellular homeostasis maintenance [78]. According to the works of literature, AMPK has a significant role in autophagy regulation through direct phosphorylating of autophagy regulators including lipid kinase complex PI3KC3/VPS34 and protein kinase complex ULK1, leading to activation of these two complexes [79]. Importantly, autophagy has a critical role in the facilitating of differentiation [80], glycogenolysis (glycophagy) [81], lipolysis (lipophagy) [82] as well as the regulation of adipose mass [80]. The discovery of the molecular association between AMPK and autophagy suggests a novel model of expanding the AMPK functional network into cellular homeostasis, like the metabolism [73].

## 4. MAPK Signaling Pathway and Autophagy

A major role in cancer progression and development is played by the mitogen-activated protein kinase (MAPK) pathway [83]. The classic MAPK pathway consists of extracellular signal-regulated kinases (ERKs), MAPK14, c-Jun NH2-terminal kinase and stress-activated protein kinase (JNK/SAPK). Mammals express at least six distinctly related groups of MAPKs including, viz. ERK1 / 2, ERK3 / 4, ERK5, ERK7 / 8, JNK1 / 2/3 and α, β, γ, and δ (ERK6) p38 isomers [84].

Three kinases evolutionarily conserved and sequentially act containing 1) MAPK, 2) MAPK kinase (MAPKK), 3) MAPKK kinase (MAPKKK) comprise each set of conventional MAPKs [85]. The MAPKKKs, as protein Ser/Thr kinases, are activated via phosphorylation and/or interaction with Ras/Rho family of small GTP-binding proteins in response to extracellular stimuli. Following MAPKKK, MAPKK is activated via phosphorylation, and it subsequently phosphorylates and stimulates MAPK activity via dual phosphorylation on Thr and Tyr residues [86].

Signaling pathways of JNK MAPK and p38 play important roles in the regulation of the balance between autophagy and apoptosis [86]. It is important to note that a vital function of p38 MAPK is related to its autophagy modulatory role in reaction to chemotherapy. As both a positive and negative controller, P38 MAPK plays two roles in regulating autophagy. First, p38 MAPK participates in autophagy, for instance, it suppresses mTOR by declining p38 MAPK phosphorylation in stomach cancer cells [87], which may result in autophagic cell death stimulated by E Platinum. Second, p38 MAPK may also negatively affect autophagy through the ULK1 phosphorylation and avoiding the binding of ULK1 and ATG13 [88]. It is important to note that p38 signaling pathway suppression leads to necroptosis and autophagy in L929 cells treated by TNFa [89].

In addition, JNK plays a vital role in autophagic stimulation in the face to stress signals. For example, it has been demonstrated that ROS activates JNK which in turn may facilitate antioxidant reactions to persuade autophagy and cell death [90]. One of the best described damage-associated molecular pattern (DAMP), called HMGB1, tends to reduce drug resistant-myeloid leukaemia cells via raising JNK-dependent autophagy. Notably in human nasopharyngeal carcinoma cells, JNK signaling plays an essential role in ceramide-induced autophagy through the LC3 up-regulation [91]. However, in head and neck cancer cells, autophagy is stimulated by bortezomib through JNK activation, which activates autophagy via two different mechanisms. First, it supports Bcl-2/Bcl-xL phosphorylation leading to the disassociation of the Beclin 1-Bcl-2/Bcl-xL. Second, the up-regulation of damage-regulated autophagy modulator is initiated by JNK [90].

## 5. Autophagy Inducer Drugs

### 5.1. mTOR Inhibitors

#### 5.1.1. Sirolimus and Its Analogues/Derivatives

Sirolimus is derived from Streptomyces hygroscopicus and is well known as rapamycin. This solid white material has a melting point between 183 and 185 °C, with a molecular weight of 914.179 Da. It is a carboxylic lactone-lactam macrolide with hydrophobic property and because of its lipophilicity feature it is considered a challenging pharmaceutic to formulate into both intravenous and oral dosage forms. Some of the effects of sirolimus will be discussed here. Initially, rapamycin was discovered as an anti-fungal medication and later its immunosuppressive and anti-tumor/anti-proliferative potential were added [92]. In brief, it is worth mentioning that rapamycin has cytotoxic effects on both dermatophytes and Candida albicans, while interestingly, the IC50 dose to affect dermatophyte is higher than yeasts. Besides its effect on non-mammalian cells, it may be applied to treat some disorders and diseases related to human cells. For example, there are some reports on the therapeutic impact of rapamycin on tuberous sclerosis complex related to autism spectrum disorder [93,94] and also reduction in the rejection of transplanted tissue in recipients through apoptosis enhancement of normal and abnormal lymphocytes [95]. However, there are additionally several studies about the positive impacts of rapamycin on multiple sclerosis and breast cancer [96,97]. Taking everything into account, there are many studies indicating the remarkable efficacy of rapamycin in the treatment or diminishing of disease and disorders. For example, it has been demonstrated that high doses of rapamycin had inhibitory effects on cancer cells while it loses its potential in cancers resistant to rapamycin. Notably, rapamycin in a dose-dependent manner affects gastric cancer cells through the induction of apoptosis. Therefore, at high concentration it exerts its anti-gastric cancer potential [98]. Surprisingly, this study showed that colorectal cancer cells have different sensitivity to rapamycin treatment. In another study, the up-regulation of mTOR and its downstream molecules was investigated and it has been revealed that the breast cancer cells are sensitive to the rapamycin [99]. Moreover, the hemangioma endothelial cells exhibit increased level of hypoxia-inducible factor 1-alpha (HIF-1α) compared to the control cells due to the promotion of VEGF/VEGF2 signaling pathway [100] while its remediation with rapamycin down-regulated the HIF-1α and VEGF-A165 and meaningfully decreases the volume of kidney tumors [101].

One of the derivatives of sirolimus is everolimus, which is a macrocyclic lactone, 40-O-(2-hydroxyethyl). It is a white to faintly yellow powder isolated from the *Streptomyces hygroscopicus* [102,103]. Notably, everolimus exhibits more bioavailability, lipophilicity potential (< 0.01% in 0.1 N HCl, water, and citrate buffer at the pH range of 2.0–10.0) and a shorter half-life than sirolimus, resulting in it more rapidly attaining a steady state [104]. 

The differences are not limited to chemical properties, but rather involve their biological activities. For example, everolimus has a higher affinity for the mTORC2 and influences mTOR signaling pathway in a different manner than sirolimus. Despite some differences in biological activities, they have some similarity in clinical applications [105]. For example, everolimus also improves long-term graft survival prior to kidney transplantation [106]. It also guarantees renal function through the renal graft survival and stable graft function [107]. Moreover, Levy et al. disclosed the efficacy of everolimus following liver transplantation. In other words, their results showed a lower rate of acute rejection in patients treated with everolimus compared to the placebo group [108]. Besides its role in the diminishing of transplanted organ failure, everolimus inhibits the growth factor-induced proliferation of hematopoietic (lymphocytes etc.) and nonhematopoietic cells derived from the mesenchymal origin. Moreover, it blocks the downstream signaling cascade of interleukin-2 receptor, leading to G1 phase cell cycle arrest [109] and inhibits intracellular signaling kinases through interconnecting to the immunophilin-FK506 binding protein 12 (FKBP12). Regarding the modulatory role of everolimus on the mTOR signaling pathway, as aforementioned, mTOR acts as negative regulator of autophagy through the diminishing of unc-51 like autophagy activating kinase (ULK1) activity [110]. To form mature autophagosomes, microtubule-associated protein 1 light chain 3 (LC3-I) is generally converted to phosphatidylethanolamine-conjugated LC3 (LC3-II). Therefore, LC3-II may be considered as a marker of autophagy activity [111]. Nakagawa et al. evaluated the level of LC3 in rat urine to assess the effect of mTOR inhibitors such as everolimus in the recovery of injured kidney [112]. Their results interestingly demonstrated the modulation of autophagy through the mTOR pathway in proximal tubular cells. Therefore, everolimus promotes autophagy through the ULK1 protein in acute kidney injury, leading to the defect regeneration of tubular cells. Besides, endocrine resistance and tumor progression are induced at the result of abnormal activation of several growth factors signaling pathways, especially the PI3K/Akt/mTOR pathway. Everolimus as the inhibitor of mTOR kinase hampers PI3K/Akt/mTOR pathway and returns sensitivity to the endocrine therapy, for instance, in hormone receptor-positive (HR+) breast cancer patients [113,114]. The mTOR pathway is responsible for the involvement of 30–40% of hepatocellular carcinoma (HCC) cases [115]. Therefore, these findings demonstrated to the efficacy of mTOR inhibitors in an immunosuppressive regimen of HCC transplant recipients. Reports related to the efficacy of everolimus in the treatment of HCC are rather controversial and necessitate deeper investigations on the efficacious of everolimus in HCC therapy. Notably, the incidence of de novo tumors following liver transplantation is between 5% to 16% and studies indicated the role of the mTOR pathway in solid tumors and lymphoproliferation disorders [116]. Taking everything into account, there is insufficient evidence related to the efficacy of everolimus on de novo tumors, notwithstanding the everolimus is administered following liver transplantation in one-third of patients in Spain [117].

Another derivative of rapamycin is temsirolimus. The U.S. food and drug administration (FDA) have approved the efficacy of temsirolimus in the renal cell carcinoma (RCC) therapy [118,119]. Temsirolimus, as a selective inhibitor of mTOR, forms a complex with FKBP-12 to inhibit the intracellular serine/threonine kinase activity of mTOR [120,121,122]. Following, the translation of major regulatory proteins involved in cell cycle progression is blocked and leads to G1/S phases arrest [123]. Furthermore, the inhibition of the mTOR pathway by temsirolimus is in line with reversed tumor-associated angiogenesis [124]. Liu et al. examined the efficacy of temsirolimus in the treatment of adenoid cystic carcinoma (ACC) through autophagy induction via the inhibition of the mTOR signaling pathway [125]. However, temsirolimus exhibits therapeutic effect for hematological malignancies and pancreatic cancer [126,127]. Besides, Kang et al. disclosed the therapeutic influence of temsirolimus and adriamycin on hepatocellular carcinoma through the apoptosis induction and enhancement in Bax/Bcl2 ratio. Their findings revealed that the combinational administration of temsirolimus and adriamycin induces higher therapeutic effect as compared to the temsirolimus or adriamycin alone [128].

In a phase 2 clinical trial study on recurrent glioblastoma patients, the efficacy of sitemsirolimus (250-mg intravenous dose weekly) was evaluated and neuroimaging results disclosed a reduction in tumor-associated T2 hyperintensity using magnetic resonance imaging [129]. It is worth noting that the combinational therapy of sitemsirolimus with bevacizumab [130] and sorafenib [131] has been shown in patients with recurrent glioblastoma.

#### 5.1.2. Dactolisib

Dactolisib—a synthetic imidazoquinoline derivative possessing hydrophobic property—is an anti-cancer therapeutic under phase I/II clinical trials. This orally administered, highly selective and reversible dual inhibitor of PI3K/mTOR has shown promising anti-solid tumor efficacy [82,132,133,134,135,136,137]. Thomas et al. revealed that the combination therapy with dactolisib and everolimus slows the progression of HCC in mice. Regarding microarray analysis, it might be said that combination therapy with dactolisib and everolimus unlike monotherapy reverts the expression level of several tumor-associated genes to the level of normal liver tissue. However, combination therapy leads to down-regulation of genes involved in autophagy [138]. Furthermore, the combination therapy with dactolisib, temozolomide (TMZ) and concomitant radiotherapy [139] showed a synergetic effect of dactolisib on apoptosis and drug efficacy in glioma cells and an orthotropic xenograft rat model [140]. Following in vitro and in vivo investigations, a phase I clinical trial has evaluated the effectiveness of combination therapy with BEZ235 and everolimus in patients with advanced or metastatic solid cancers. However, limited tolerance to combination therapy prevented dose escalation to where efficacy could potentially be achieved [141]. Moreover, combination therapy with dactolisib and abiraterone acetate (AA) in phase Ib clinical trial was discontinued in patients with castration-resistant prostate cancer due to safety and tolerability concerns [142].

### 5.2. AMPK Activators

Any modulator that leads to calcium and AMP accumulation triggers AMPK. The activators of AMPK are classified into directly and indirectly activators. In this study, two AMPK indirect activators are discussed [73,143].

#### 5.2.1. Metformin 

Metformin, an antidiabetic drug, is one of the AMPK activators. It is a synthetical biguanide derived from guanide that is extracted from *Galega officinalis* plant. An outstanding role of metformin is diminishing of hepatic glucose production and enhancement of peripheral insulin sensitivity [144]. Notably, Owen et al. discovered the molecular mechanism behind the antidiabetic actions of metformin by AMPK. They demonstrated that metformin performs antidiabetic action through the stimulation of glucose uptake, hepatic glucose production, fatty acid oxidation and down-regulation of lipogenic genes [145]. Interestingly, it has been shown that blood glucose levels reduced by metformin in the animal models of LKB1 knockout and liver-specific AMPKα knockout [146].

In agreement with the anti-cancer property of metformin in vitro and in vivo, several epidemiological and meta-analyses studies revealed the lower cancer risk in diabetic patients treated with metformin compared to non-metformin theraputics, demonstrating an association between metformin and anti-cancer potential. The promising outcomes from the in vitro and in vivo experiments attracted the widespread attention to elucidate the mechanisms of metformin in the context of cancer prevention. It seems that the main targets of metformin that exert its antitumor impacts are complex I in the mitochondrial electron transport chain (ETC), AMPK and mTORC1 [147,148,149]. These findings approve the findings of Rabiee et al. in which they disclosed that autophagy inducers are a promising candidate in cancer therapy at the onset of cancer while they will not be effective in the later stages [150]. 

#### 5.2.2. Simvastatin 

Another AMPK activator is simvastatin. It belongs to the statin drugs [151] and indirectly activates MAPK and is adminstrated as an effective drug in obesity treatment and lowering of the risk of cardiovascular diseases. Lately, a considerable amount of attention has been paid to existing drugs that are administrated chronically in the treatment of hypercholesterolemia and hypertriglyceridemia. Among these drugs, simvastatin is the most frequently used drug despite its poor solubility in water. 

Multiple reports have documented the antineoplastic potential of statins in a variety of cancer. A systematic review demonstrated the beneficial effects of statins in overall survival and cancer-specific survival [152]. Although the findings of epidemiological studies do not match the relationship between statins and the risk of cancer for various cancers [152]. The precise molecular mechanism by which statins exert anti-cancer activity is not fully understood. Unspecified mode-of-action (related to AMPK activity) is possibly acting as a mitochondrial poison [153]. Moreover, blocking Shh signaling [154], increasing the amount of RhoA-GTP, inhibiting the interaction between RhoA and Rho-GDI [155], inducing the activation of AMPK and p38 MAPK [155] are suggested to be implicated in anti-cancer effects of simvastatin. Furthermore, Wei et al. reported that simvastatin leads to diminishing of mTOR activation, LC3B and Beclin1 up-regulation and autophagosomes-lysosomes fusion to induce autophagy [156].

### 5.3. MAPK Activators

MAPK pathway is activated in part via extracellular stimuli including genotoxic agents, oxidative stress, ultraviolet irradiation and Gi-Coupled Receptor (GPCR). However, cell growth, cytokine stimulation and inflammatory factors can also result in the induction of the MAPK pathway. Activated MAPK has a vital contribution in the signal transduction of extracellular stimuli into cells to an ample amount of cellular responses for example differentiation, proliferation, senescence, and so on [157].

#### Carbamazepine 

Carbamazepine (CBZ), trademark Tegretol, is a dibenzaepine derivative that is widely prescribed in patients suffering from epilepsy and neuropathic pain to diminish various types of seizures. It is noteworthy that oral administration exhibits poor bioavailability, partly owing to poor water solubility. Although, drug absorption from IR dosage forms is slow and erratic, chronic usage and overdose exhibits serious side effects that it may suggest ER system involvement. It triggers autophagy through changing the levels of myoinositol-5,4,1-triphosphate [158]. Along with other medications, carbamazepine is prescribed to cure schizophrenia as a second-line medication in bipolar disorders. It blocks sodium channels through the binding to voltage-gated sodium channels while in an inactive conformation, and inhibits insistent and continuous raising of an action potential [159].

## 6. Autophagy Inhibitors: Main Agents and Mode of Action

Malignant cells in parallel to healthy cells benefit from the cytoprotective effects of autophagy. There are many reports and efforts on the efficacy of autophagy inhibitors in cancer therapy and more efforts to find and design specific drugs with autophagy inhibitory potential. However, there is controversy on the effectiveness of autophagy inhibitors in cancer therapy and some reports have related its efficacy to the stage of cancer progression [150].

**Figure 2:**
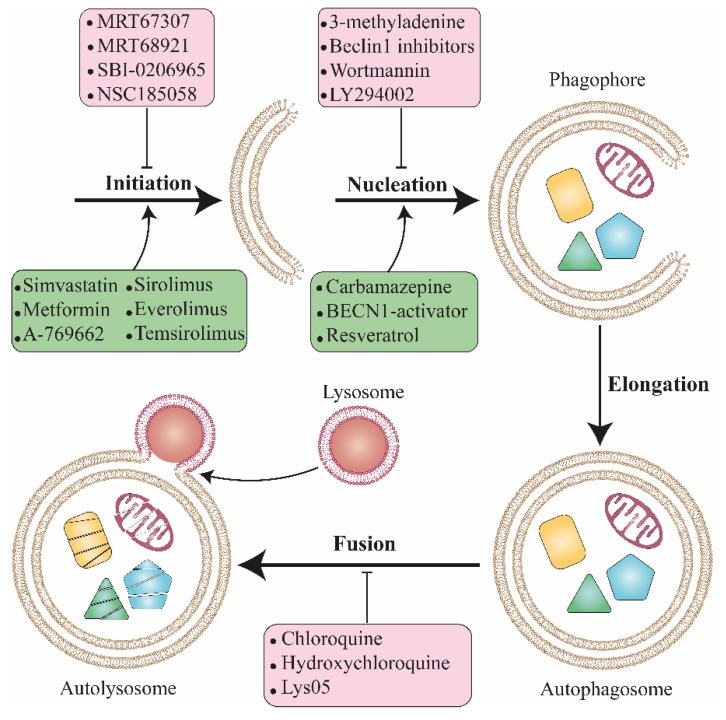
Schematic presentation related to the effect of different drugs on the various stages of autophagosome formation. There are some hot points in autophagosome formation including initiation, nucleation, elongation and fusion. However, various biomolecules and drugs influence these stages and may modulate autophagy to enhance cell survival or mortality in healthy and injured cells.

Autophagy is a druggable process that can be pharmacologically targeted at several step points (Figure 2) [160]. It is often stimulated in a malignant cancers context and subsequently plays a key role in tumorigenesis, tumor resistance and evasion based on a different stage of cancer progression [161]. Therefore, there is intense interest to improve novel therapeutic agents to harness autophagy where it enhances cancer cell survival. In 1998, Murakami et al. [162] reported that chloroquine (CQ) is able to block autophagy and Briceno et al. [163] demonstrated that CQ retains antitumor activity in glioblastoma in 2003. The results of phase I/II clinical trials using hydroxychloroquine (HCQ) show the anticancer effect of this drug in cancer patients [164,165]. Prior to autophagy modulation, the following questions should be addressed. Autophagy inhibition is effective in which stage of cancer progression? What degree of selectivity through autophagy pathway is ideal to inhibit with therapeutic agents? In the autophagy pathways, which steps are optimal to intervene pharmacologically to modulate autophagy machinery? What will be the fate of other signaling pathways associated with autophagy process in targeted cells?

### 6.1. Autophagosome Degradation Blockers

Chloroquine, *N*′-(7-chloroquinoline-4-yl)-*N*,*N*-diethyl-pentane-1,4-diamine, is a 4-aminoquinoline (also known as CQ phosphate) was synthesized by Andersag in 1934 and it was introduced into a clinical trial in 1947 against malaria [166,167]. In 1946, Surrey et al. developed HCQ sulfate. They claimed that the addition of a hydroxy group into one of the N-ethyl groups of CQ phosphate would intensively decrease its toxicity [168,169]. In addition to antimalarial activity, HCQ sulfate has an anti-rheumatic effect [170,171]. CQ analogues are recognized as lysosomotropic agents and hamper lysosomal acidification, which in turn hinders proteolysis, autophagosome degradation, chemotaxis, phagocytosis and antigen presentation [172]. The autophagic cargo is degraded and the degradation products eventually reach the cytosol via lysosomal permeases; thus, they enter the recycling process in biosynthetic metabolic stream [173]. It is noteworthy to mention that lysosomotropic agents suppress autophagosomes fusion with lysosomes and their biodegradation [174]. 

Recently, CQ analogues have been studied for their potential anticancer activity. However, anticancer potential of CQ and HCQ is mainly through the modulation of signaling pathways other than autophagy. For example, CQ has recently been demonstrated for targeting cancer stem cells through inhibition of Janus kinase 2 (JAK2) signaling pathway [175]. These lysosomotropic compounds are definitely very proficient to induce lysosomal membrane permeabilization and initiate mitochondrial apoptosis [176]. Notably, CQ is the only autophagy inhibitor with FDA approval.

### 6.2. Class III PI3K Inhibitors

To shed light on the role of class III PI3K in autophagy, it could be said that autophagy starts with the activation of the ULK1 (also identified as ATG1) complex (ULK1, ULK2, FIP200, ATG13 and ATG101). The ULK1 complex leads to vesicle nucleation by means of class III PI3K complex including UV radiation resistance-associated gene protein (UVRAG), VPS34 and ATG14. Notably, there are three types of PI3K in mammals. In brief, whereas PI4,5-bisphosphate is the main target for phosphorylation by class I PI3K and generation of phosphatidylinositol 3,4,5- trisphosphate (PI(3,4,5)P3), class III PI3K/hVps34 mostly phosphorylates phosphatidylinositol [21] to produce phosphatidylinositol 3-phosphate (PI3P). There are a few reports regarding the function of class II PI3K and it seems that it catalyzes phosphorylation of PI to PI3P and PI 3,4-bisphosphate [177]. 

As mentioned above, autophagy promotes in part through class III PI3K and the first mechanistic understandings of the autophagy machinery came back to the discoveries of autophagy inhibitors like 3-methyladenine (3-MA) by Seglen and Gordon [178] in 1982. However, later Blommaart et al. discovered that 3-MA inhibits class III phosphatidylinositol-3 kinase [179] and it also serves as kinase and phosphatase blocker [180]. Notably, 3-MA plays a dual role in autophagy. From one side, it enhances autophagy when treated in complete medium for a long time, while from the other side in the face of starvation it suppresses autophagy. Therefore, based on their ability to inhibit class III PI3K, 3-MA and wortmannin can be extensively used as inhibitors of autophagy [181]. Furthermore, the autophagy induction activity of 3-MA is not only owing to the class III PI3K but also, is owing to the temporal effect on class I. In other words, even though 3-MA inhibits class I PI3K persistently, its inhibitory effect on class III PI3K is reversible. Considering the controversial role of 3-MA in autophagy, attention should be paid in the administration of 3-MA as an autophagy modulator [181].

It is worth noting that wortmannin, a steroid metabolite of the fungi *Penicillium funiculosum*, is capable of inhibiting autophagy no matter what the nutrient status. It has similar affinity for three classes of PI3K (I, II, and III) in vitro. Furthermore, it can hinder other PI3K-related enzymes including MAPK, mTOR, myosin light chain kinase (MLCK) and some phosphatidylinositol 4-kinases at high levels [182,183,184]. Importantly, wortmannin failed in its clinical translation because of its high toxicity, poor solubility and low stability.

## 7. New Therapeutic Agents that Target Different Steps of the Autophagic Machinery

Studies are only just beginning to find and develop a novel small molecule to inhibit the autophagy and may open a new horizon to selectively targeting this process [185]. Several preliminary types of researches are worth reviewing as follows.

### 7.1. Targeting ULK1/2

SBI-0206965 (2-((5-Bromo-2-((3,4,5-trimethoxyphenyl)amino)pyrimidin-4-yl)oxy)-*N*-methylbenzamide) is the serine/threonine autophagy-initiating kinases ULK1 and ULK2 selective inhibitor, however, it has high selectivity for ULK1 [186]. Besides, it blocks the phosphorylation of Beclin-1 and VPS34. SBI-0206965 enhances apoptosis in cancer cells through the diminishing of autophagy following mTOR inhibition. 

Another small molecule involved in ULK1 inhibition is MRT67307 or MRT68921. They reduce the amounts of ATG13phosphorylation at serine 318, a characterized phosphorylation site for ULK1 [187]. Treatment with both MRT67307 and MRT68921 (*N*-[3-[[5-Cyclopropyl-2-[[3-(4-morpholinylmethyl)phenyl]amino]-4-pyrimidinyl]amino]propyl]cyclobutanecarboxamide dihydrochloride) decreases autophagosome maturation through the conversion decrement of LC3-I to the LC3-II [188].

### 7.2. Targeting Ubiquitin Specific Peptidases (USP)

Specific and potent autophagy inhibitor-1 (spautin-1 or Spautin-1 (6-Fluoro-*N*-[(4-fluorophenyl)methyl]-4-quinazolinamine) suppresses autophagy through the inhibition of USP13 and USP10. They typically target the Beclin 1–Vps34 complex, leading to increased degradation of the Beclin 1–Vps34 complex [189].

### 7.3. Targeting Autophagy-Related Protein 4B (ATG4B)

ATG4B through its protease function cleaves key protein located in autophagosome membrane (C terminus of proLC3). Absence of the ATG4B protease leads to a decrease in LC3-II-driven autophagosomes maturation [190]. It has been reported that NSC185058 (N-pyridin-2-ylpyridine-2-carbothioamide) inhibits ATG4B, resulting in inhibition of LC3B lipidation and autophagy maturation without disturbing the mTOR or PtdIns3K pathways [191].

### 7.4. Targeting p62

p62 is another modulator of autophagy and its level is an important marker of autophagy function and dynamic. It is an autophagy receptor that binds to the cargos (with polyubiquitin) and interacts with LC3 to aid the engulfment of the cargos by autophagosome. P62 level is inversely proportional to the amount of LC3B. However, in dysregulated autophagy p62 will decreased in parallel to LC3B [192]. Interestingly, it was the first discovered autophagy modulator in mammals [193,194]. p62 was named sequestosome 1 (SQSTM1) by Shin owing to its ability to form aggregates [194]. Recently, p62 was also shown to transport ubiquitinated proteins like tau, to the proteasome. Moreover, it can shuttle between the cytoplasm and nucleus to bind with ubiquitinated cargos and facilitate quality control of nuclear and cytosolic proteins [195]. Verteporfin, an approved agent used in photodynamic therapy of age-related macular degeneration, is a benzoporphyrin derivative and inhibits autophagy through p62 in absence of light activation [196]. Donohue et al. showed that combination therapy of verteporfin with gemcitabine moderately increases chemotherapeutics efficacy in a pancreatic ductal adenocarcinoma mode [197].

## 8. Autophagy Inhibition for Cancer Therapy 

The role of autophagy in cancer is a double-edged sword [198] and depends on several parameters, including stage and type of cancer and genomic context. There are several pieces of evidence highlighting the protective effects of autophagy on tumor prevention [6,15]. Previous studies maintained that the elimination of beclin1 gene is associated with ovarian, breast, and prostate cancer. Additionally, aggregating p62 resulted from autophagy suppression, leading to cellular toxicity, oxidative stress, and DNA damage. Furthermore, UVRAG and Bif-1 positively regulate autophagosome formation and disruption of these genes was detected in different types of cancers, including colon, gastric, breast, prostate, and bladder cancers [16]. However, in developed tumors, autophagy can give rise to chemoresistance and cancer cells survival by mitigating cellular stress and providing nutrient [199]. Taken together, autophagy induction is beneficial to the protection of normal cells and hampers tumor initiation. However, autophagy inhibition is in favor when treating advanced cancers, and exceeding activity of autophagy can synergistically give rise to apoptosis [15,20].

Rabiee et al. unveiled that cancer therapy ought to be considered at two stages of initiation and progression, in which at the initial stage autophagy inhibitor medication would not be effective, versus apoptosis inducers, while at the progression stages, autophagy inhibitors would be crucial [150]. Surprisingly, cancer cells due to Warburg effect and ammonia as the by-product of glutaminolysis exhibit enhanced autophagy [200,201]. In fact, autophagy acts dually in cancers. In wild type p53 tumors autophagy diminishes tumor progression while in tumor with P53 mutation it increases cancer progression [202,203]. In other words, autophagy has been generally considered as a cytoprotective (pro-survival) mechanism. However, its imbalance may lead to the pathologic condition. At present, at least four different functional forms of autophagy have been defined [204]. (1) Cytoprotective role: when autophagy inhibits cells death or arrest; (2) Cytotoxic: when autophagy induction leads to cell death and its inhibition leads to cell survival; (3) Cytostatic: when autophagy leads to arrest of cell growth and (4) Nonprotective: when autophagy suppression does not affect cell growth [205]. 

The crucial step in designing an effective therapeutic approach is to understand the correct and accurate causes of diseases and to understand whether autophagy plays a cytoprotective or cytotoxic/cytostatic roles. It will help scientists to find approaches to manipulate tumor cells and pathologic cells in order to enhance cellular sensitivity to therapeutics and treat them. Targeting of the cytoprotective arm in autophagy is the key basis for many clinical trials. Certainly, if enhanced autophagy leads to tumor resistance to death-inducing agents, its inhibition confers a boosted response to treatment [206].

A dozen phase I and I/II clinical trials have been carried out and published based on the autophagy blockers such as CQ [207] or HCQ in cancer, over the last decade [208]. An efficient and safe combination therapy with HCQ, antimetabolite and gemcitabine in phase I/II clinical trial was performed in pancreatic cancer patients [164]. The combination of HCQ, histone deacetylase inhibitor and Vorinostat conducted in patients with metastatic colorectal cancer and found that five out of 19 patients achieved prolonged stable disease [209]. Similarly, a dose-escalating study in melanoma with combination therapy of HCQ and temozolomide showed that 14 of the 17 patients maintained stable disease [165]. However, there are several ongoing clinical trials on tyrosine kinase inhibitors (TKIs) combined with autophagy inhibitors as a chemotherapeutic agent in cancer therapy with the aim of pervasive targeted therapy [210,211]. For example, chronic myeloid leukaemia (CML) stem cells are naturally resistant to second/third-generation tyrosine kinase inhibitors (TKIs) and Calabretta et al. reported that inhibition of autophagy via CQ eliminated CML-enriched stem cells [212]. For the rest of the review, we discuss the encapsulation of autophagy modulators into nanocarriers to improve their efficacy, solubility and bioavailability. 

## 9. Nanostructures for Autophagy Modulator Delivery Systems

Deemed one of the major reasons for mortality in the young and elderly population, cancer remains incurable today. This is to some extent because we lack understanding of its special mechanisms, and because the triggering of different signaling pathways in cancer cells makes cancer therapy more complicated. Interestingly, nanomedicine is promising for cancer therapy since the sensitivity of cancer cells to some NPs is higher than normal cells and therefore, make NPs a valuable candidate in passive tumor targeting [40,213]. As mentioned earlier, recent studies indicated that autophagy has an important role in the modulation of tumor stage and neurodegenerative disorders. Despite a great deal of investigation already in the field neurodegenerative disorders, there has recently been a marked increase in research in this area [214,215,216,217,218,219,220]. Among the main challenges in drug delivery are drug non-selective biodistribution, hydrophilicity, and cell uptake. To overcome these obstacles, it appears that encapsulation of drugs into the nanocarriers may be efficacious [213,221,222]. Nanoparticles (NPs) act as a foreign biomolecule in body and are in the size range of viruses and some small bacteria, therefore, they can provoke cells to enhance autophagy [192]. Notably, the size of NPs and carriers influence their biological activates [223,224,225,226,227]. To sum things up, encapsulation of autophagy modulators into nanocarriers is recommended as a promising approach to tackle this challenge (Table 1).

**Figure 3:**
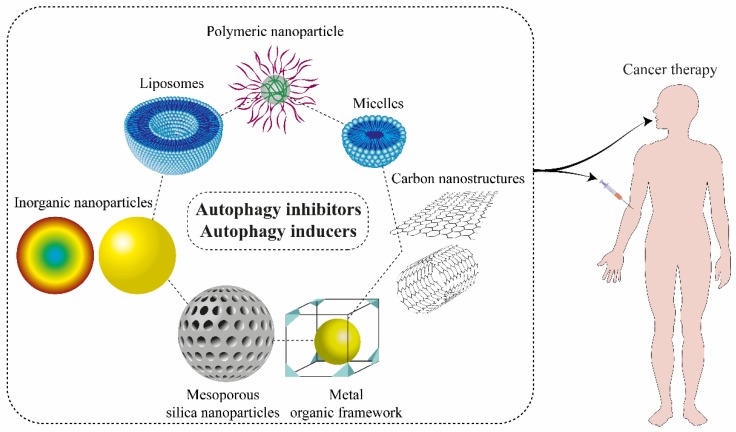
Various nanocarriers for delivery of autophagy modulators.

The use of nanoparticles as autophagy modulator carrier carries several advantages such as high delivery efficacy, low systemic toxicity, and prevention of drug resistance [228,229]. Despite the usefulness of utilizing more common carriers included liposomes, micelles, and polymeric nanoparticles for autophagy medication (Figure 3), some researchers suggest that employing metal nanoparticles like silver, gold, and iron oxide either alone, in combination with autophagy drug suppressors or with an external inducer (like laser exposure) can result in excessive autophagy inhibition. Moreover, a few studies focused on other nanomaterials like nanogels, molybdenum disulfide (2D nanosheets), and lipid calcium phosphate nanoparticles on cancer therapy [34].

Nanostructures are human-made structures with at least one dimension in approximately 1 to 100 nanometer [12] and they have different chemical-physical properties than balk materials [272]. Nanomaterials may have 1, 2 and 3 dimensional including nanosheets (graphene oxide), nanofibers [223,273,274,275], carbon based NPs [276,277], quantum dots, polymeric, ceramic NPs, liposomes, micelles [278], metalic NPs [279] etc., respectively. Regarding Wilhelm et al., active targeting of rod shape inorganic nanocarriers with the particle size of less than 100 nm, neutral zeta potential to solid tumors exhibits significantly higher drug delivery efficacy than organic, positive or negative zeta potential nanocarriers [280]. As mentioned above, to overcome poor solubility, low stability, and targeted bioavailability, nanocarriers encapsulation of autophagy modulators can renew their clinical translation potential [281]. 

Chen et al. designed a metal-organic framework (MOF) NPs containing 3-MA as chemotherapeutics agent [269]. The autophagy inhibitory mechanism of 3-MA is through the inhibition of the class III PI3K (Vps34)/Beclin-1 complex that leads to prevention of autophagosome formation. Notably, their results indicated that high levels of 3-MA encapsulation (19.798 wt%) significantly blocks the formation of autophagosome compared to the free 3-MA and it also has superior cytotoxic impact on HeLa cells in a dose-dependent manner. In another study, Shi et al. [270] fabricated a pH-responsive and tumor-targeted zeolitic imidazole NPs (ZIF-8) containing CQ. To enhance drug uptake of NP, they were decorated with methoxy poly (ethylene glycol)-folate (FA-PEG). These findings showed superior cell mortality of cancer cells through the autophagy blockage. 

### 9.1. Liposomes

Liposome is considered to have been the first commercialized drug nanocarrier for the cancer therapy in 1995. However, it is extensively administrated for the treatment of many diseases [213,282]. The pH- and temperature- dependent features of liposomes make it favorable for tumor recognition and targeting especially in photothermal therapy [243,283]. Regarding liposome constituents, it exhibits biocompatibility, diminishes drug biodegradation improves drug solubility and target-specificity, resulting in liposome being considered a favorable drug delivery system [284]. Ghanbarzadeh et al. synthesized a pH-sensitive and plasma stable liposome containing monomethyl itaconate as a lipid base and rapamycin as an autophagy inhibitor to cancer therapy. Their results demonstrated that liposomes containing rapamycin are more efficient and have more cytotoxicity and inhibitory effect compared to the rapamycin alone [285]. Yang et al. examined the efficacy of PEGylated liposomes containing metformin and epirubicin against CD133^+^ cancer stem-like cells. Their data showed that liposomes-encapsulated metformin and epirubicin have higher cytotoxicity compared to the metformin and epirubicin alone [286]. Alupei et al. prepared liposome containing simvastatin to examine its inhibitory effect on tumor growth [287]. They used B16.F10 melanoma tumors and showed that liposomes containing simvastatin remarkably inhibit the growth of tumor through blockade of the intratumor generation of HIF-1α.

Beside the role of liposomes in passive targeting, it may apply as an active targeting through the binding with ligands such as antibodies. For example, Gholizadeh et al. [234] prepared a decorated E-selectin antibody liposomes for targeted rapamycin delivery to TNF-α activated cells. They hypothesized that the system will decrease the side effects of the rapamycin on other cells.

### 9.2. Micelles

Micelles are regarded as the other types of nanocarriers and are synthesized in a high and low energy consuming manner. Micelle preparation is based on the amphiphilic co-polymers and surfactants with various hydrophilic-lipophilic balance (HLB) [213,230]. Based on the type of micelle in which they are oil-in-water or water-in-oil, the hydrophobic and hydrophilic sections will be on the core or shell respectively. The preparation process of micelles is convenient and the changeable feature of core and shell in micelles exerted micelles with multi-functional and stimuli-responsive properties. Notably, micelles exhibit higher solubility, stability and biodistribution than the conventional drugs; therefore, these properties make them a desirable choice for the encapsulation of autophagy inhibitors. Chen et al. prepared micelles containing rapamycin for cancer therapy. They treated HCT 11b and HeLa cells with rapamycin-micelles and demonstrated that rapamycin-micelles have more cytotoxicity effects on the viability of cells compared to the rapamycin alone [288]. Furthermore, Shaki et al. showed that pH-sensitive micelles containing rapamycin have a stronger cytotoxic effect than rapamycin on glioblastoma multiform (U87 MG-cell line) [289]. Liu et al. prepared a micelle containing simvastatin using membrane dialysis method and investigated its effect on human osteoblast-like MG-63 cells. Their result demonstrated that simvastatin releases in a prolonged manner and thereby induces the enhanced differentiation and mineralization of osteoblast through the BMP-2 pathway [290]. Therefore, micelles containing simvastatin may be considered as a candidate in fracture healing [291]. 

### 9.3. Polymeric Nanoparticles

Biodegradable polymers NPs are considered to be the important nanoplatform for drug delivery applications [292]. The polymeric NPs exhibit high stability and release the drug in a controlled manner mechanism along with high entrapment efficacy [293]. Based on the constituents in liposomes and polymeric NPs, they exhibit different biological impacts including biocompatibility. However, some reports indicated that liposomes have a narrow application because of unregulated release, instability in storage and inadequate drug loading [294]. Polymer-hybrid lipid NPs or polymerosomes benefit from the advantages of both liposomes and biodegradable polymers, whereas they may not have the disadvantages of liposomes and biodegradable polymers. They release the drug in a controlled procedure, with high biocompatibility and desirable pharmacokinetic traits. Li et al. synthesized a polymer-lipid NP containing rapamycin and investigated the effect of the nanocarriers in mice suffering from subcutaneous hemangioma. Their results showed that nanocarriers significantly decrease the viability of mice bearing hemangioma compared to the rapamycin alone [235]. 

Besides polymer-lipid NPs, polymeric NPs may be also considered in cancer therapy. For example, Dactolisib can be delivered into a target site using pegylated PLGA NPs with high cell up-take potential, and this makes them an ideal candidate in drug delivery [271]. Moreover, Kasper et al. synthesized a modern nanocarrier for topical delivery of everolimus [268]. They prepared a nanostructure based on methoxy-poly (ethylene-glycol)-hexyl substituted poly (lactic acid) (mPEGhexPLA) and then loaded everolimus into the nanocarriers. Interestingly, topical nanocarriers administration prevents T cells infiltration while causing a few adverse side effects on the immune response of spleen. In another study, Ding et al. prepared thermoresponsive nanocomposite gel to inhibit the growth of glioma through autophagy induction. They treated C6 cells with the nanocarriers and showed that these cells are more sensitive to paclitaxel compared to the temozolomide and notably its combinational therapy induced higher cytotoxic effects through the autophagy induction. Besides this, they also showed that nanocomposite gel promotes anti-glioma tumor effects via local drug delivery [295]. Shi et al. [296] designed bovine serum albumin conjugated carboxymethyl-beta-cyclodextrin NPs laded with gefitinib. The folate decorated NPs used to enhance drug delivery. The nanocarriers reduce autophagy through the LC3 protein down-regulation and thereby increase apoptosis in folate receptor-positive HeLa cells through the inhibition of ATP synthesis and up-regulation of caspase-3 protein. Song et al. evaluated the role of autophagy during intracellular random siRNA delivery via lipoplex and polyplex NPs [297]. It was shown that autophagy was independent of mTOR pathway. Furthermore, the efficacy of siRNA knockdown can be remarkably increased or blocked with a variety of autophagy regulators and consequently changes the intracellular delivery. Therefore, autophagy modulators may be potential candidates for targeting gene silencing. In another study, Zhang et al. prepared a PLGA NPs to evaluate the impact of autophagy inhibitors on drug delivery in cancer treatment. Results indicated that autophagosomes seize the endolysosome-escaped NPs and guide them to the lysosomes for biodegradation, guaranteeing the effectiveness of NPs encapsulating autophagy inhibitors of 3-MA and CQ [298] in intracellular drug delivery. Cancer cells intelligently promote autophagy as a pro-survival mechanism to increase chemotherapeutic resistance. Therefore, it might be said that PLGA NPs containing autophagy inhibitors are beneficial for an increase in therapeutic effects [298]. 

## 10. Co-Delivery of Autophagy Inducers/Inhibitors and Chemotherapeutics/siRNA

Accordingly, to design an efficient drug delivery system with promising outcomes in cancer therapy, it is vital to know the phase of cancer, mutation, and heterogeneity in signaling pathways, and mechanisms involved. It is intriguing that both rapamycin and even ATP-competitive inhibitors of mTOR induce drug resistance which results from the inducible mutations in mTOR. In any case, there are several cancers that intrinsically have mTORC1 mutations [51]. Eminently, moderately successful combination therapy was observed with rapalog (everolimus) and pazopanib (tyrosine kinase inhibitor) [299]. Taglieri et al. reported that drugs that inhibit survivin, reverse the anti-tumor potential of everolimus [300]. More than 10 clinical trials have now been completed or are still running in phase I/II with FDA approved autophagy inhibitors CQ and HCQ alongside commercial drugs in multiple solid tumors treatment including pancreatic cancer, glioblastoma, and astrocytoma, prostate cancer, small and non-small cell lung cancer, refractory or relapsed multiple myeloma, breast cancer, colorectal cancer, etc. [15]. At the same time, a good deal of preclinical evidence suggests that simultaneously mTOR inhibition along with autophagy suppression could boost cytotoxicity in tumor cells. In a recent report, phase I/II clinical trial of combined everolimus (mTOR inhibitor) and HCQ (autophagy inhibitor) was administrated orally in 38 patients with clear cell renal carcinoma who previously received 1–3 VEGF-TKI regiment. From trial I, oral everolimus 10 mg daily and oral HCQ 600mg twice daily had chosen as optimal dose. Although findings indicated that <10% of patients experienced grade 3–4 adverse events, recommended drug dose was well tolerated, and the majority of adverse events was grade 1–2 including nausea, fatigue, anemia, diarrhea, and rash. HCQ did not exacerbate everolimus toxicity, and combination therapy was well tolerated and reached >40% PFS at 6.3 months. However, inducing mutations in the mTOR signaling pathway contributed to decreased PFS in this regiment [29].

**Figure 4:**
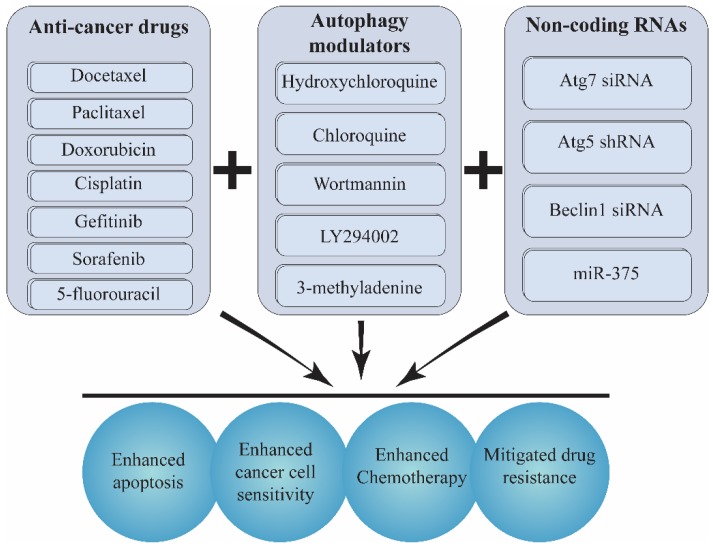
A combination of chemotherapeutic agents with autophagy genes modulators or autophagy regulators enhances the potential of chemotherapy.

Accordingly, numerous studies support the efficacy of combination therapies by incorporating a conventional chemotherapy drug like docetaxel, paclitaxel, doxorubicin (DOX), cisplatin, and 5-fluorouracil with an autophagy inhibitor such as LY294002, wortmannin, CQ and small interfering RNAs for promoting chemotherapy and reverting drug resistance [301,302] (Figure 4). 

For instance, using docetaxel in the long term could positively regulate autophagy, which eventually leads to chemoresistance and tumor survival. Recently Zhang et al. loaded siAtg7 and DTX into iRGD modified Pluronic P123-PEI co-polymer micelles for in vitro and in vivo evaluation. iRGD peptide enhanced drug accumulation and penetration in animal studies. Co-treatment with siAtg7 and DTX improved therapeutic potential by effective siRNA silencing of Atg7 and subsequent suppression of LC3 which leads to down-regulated autophagy induced by DTX in pancreatic cancer cells and PANC-1 xenografts mice model [21].

Furthermore, to overcome the insensitivity of the hypoxic regions of tumors to mTOR inhibitors, the combination of RNA interference or acetazolamide (carbonic anhydrases inhibitor) with mTOR inhibitors such as rapamycin has been proposed [52]. Interestingly, the theory of insensitivity to mTOR in the face to the hypoxic condition is consistent with acidic pH microenvironment of cancer cells. The tumor microenvironment is acidic and the environment dictates different signaling pathways through the upstream signal transduction pathways [303]. Cancer cells in the face of acidic pH stimuli decrease mTORC1 activity and lose the anti-proliferative potential of mTOR inhibitors [33,304]. To overcome this effect and increase the anti-proliferative effect of mTOR inhibitors in the face of acidic tumor microenvironment, the alkalization of tumor microenvironment with sodium bicarbonate has been recommended [33]. Taking everything into account, designing a combination therapy with an autophagy inducer system independent of mTOR is preferred. However, Conciatori et al. [305] proposed a combination therapy of everolimus and exemestane in breast cancer patients with hormone-dependency. 

As mentioned earlier, another signaling pathway that triggers autophagy is MAPK activation. Although acidity does not influence MAPK activity [33], there are some reports regarding the anti-tumor resistance of MAPK and its phosphatase (MKPs) as an example resistance to tamoxifen, paclitaxel, DOX, and mechlorethamine [306].

Fasting is another trigger for autophagy and recent reports disclosed that fasting increases the chemotherapy efficacy of anti-tumor medications [307,308] and also decreases the side effects of chemotherapy and stress resistance [309]. It is unlike the prescription of American Cancer Society related to the high caloric and protein intake in cancer patients [310]. Notably, fasting protects normal cells from the lethal effects of chemotherapy and induces tissue regeneration in healthy cells while it increases the chemotherapy efficacy on cancer cells. For instance, Groot et al., reported that the short term fasting in HER2 negative breast cancer patients inhibits dropping of RBC and platelet decrement and also in part DNA damage [311].

Besides combination therapy with autophagy inducers, there are several reports on combination therapy with autophagy inhibitors. Among the many pre-clinical autophagy inhibitors, HCQ has an FDA approval [312]. It inhibits autophagy through lysosomal acidification and thereby inhibits autophagosome degradation [313]. In the present review, we discuss the combination therapy with autophagy inhibitors in preclinical studies and HCQ in clinical trials. It is worthy of note that HCQ has modest autophagy inhibitory in high dose and also loses autophagy inhibition in acidic pH [314]. 

There have been some pre-clinical investigations on combination therapy with autophagy inhibitors. It is important to know whether drug-free NPs stimulate autophagy system or not. An investigation by Zhang et al. disclosed that PEGylated PLGA micelle induces autophagy and can also be degraded in autophagosomes [315]. Gong et al., demonstrated a successful combination therapy containing Atg7 siRNA crosslinked to docetaxel into a micellar formulation for breast cancer therapy on MCF-7 cell line. Their results showed enhanced apoptosis and autophagy inhibition in cancer cells and tumor inhibition in mice, in part owing to entrapment into the tumor site [316]. Another report on the inhibition of Atgs is related to short hairpin RNA-expressing plasmid DNA that silence Atg5 as an arm of autophagy [317]. Zheng et al. prepared a combination therapy system containing shRNA Atg5 and gefitinib into a chitosan NPs as a nanocarrier. Their findings indicated in the enhanced bioavailability of nanocarriers along with increased apoptosis and inhibited autophagy in PCL and A549 cells. However, the combination therapy with nanocarriers had diminished the tumor volume compared with naked combination therapy [317]. Notably, the encapsulation of Sorafenib (chemotherapeutic in hepatocellular carcinoma (HCC)) and miR-375 (autophagy inhibitor) into lipid coated calcium carbonate NPs increases cell mortality of HepG2 cells as compared to the Sorafenib and also, combination therapy significantly diminishes the tumor volume [318]. Notably, the combination therapy inhibits autophagy derived from chemotherapy with Sorafenib [318]. Moreover, LY294002 is an autophagy inhibitor that targets PI3K. Saiyin et al. prepared a combination therapy containing LY294002 and DOX into a polymeric hyperbranched polyacylhydrazone micelle. They demonstrated that combination therapy increases apoptosis and autophagy inhibition in HN-6 and CAL27 cells. Interestingly, they showed that combination of LY294002 into one nanocarrier enhances apoptosis and autophagy inhibition in cancer cells compared with the separately administrated LY294002 plus nano DOX [319]. 

To consider the only autophagy inhibitor with FDA approval, we followed up the investigations related to combination therapy with HCQ. Zhang et al. demonstrated that combination therapy with HCQ and Docetaxel encapsulated into a PEGylated PLGA micelle resulted in enhanced cell mortality of MCF-7 cells along with diminishing tumor volume in e SCID mice bearing MCF-7 cells [315]. Yin et al. disclosed that combination therapy with HCQ and paclitaxel encapsulated into liposome on melanoma cells resulted in metastasis inhibition through MMP2 and 9 down-regulation and tumor growth inhibition in part due to higher retention in tumor site [320].

Besides pre-clinical investigation, to survey the combination therapy in human and clinics, some valuable reports will be discussed. O’Hara et al., in a clinical trial phase I in patients with metastatic pancreatic adenocarcinoma prescribed HCQ (600 mg/w) in combination with gemcitabine/nab-paclitaxel (NCT01506973). Their results indicated in the autophagy inhibition and the good toleration of patients with combination therapy [321]. Following, in a clinical trial phase II, they showed that combination therapy did not increase the rate of survival in metastatic cancer patients [322]. Sotelo et al. investigated the combination therapy with CQ (150 mg/d) and conventional chemotherapy in patients with glioblastoma multiform. Although the rate of survival was higher in combination therapy than monotherapy, it was not significant [323]. Vogl et al. demonstrated that combination therapy with HCQ and bortezomib in patients suffer from myeloma, in which it leads to 45 % stability of disease [324]. In another study, with the combination therapy of HCQ and temozolomide in melanoma patients, it was shown that the combination therapy is well tolerated in patients [165]. Poklepovic et al. surveyed on clinical trials of combination therapy. They indicated that it is not clear whether the combination therapy with HCQ (600 mg/d) and temozolomide, vorinostat, bortezomib, and temsirolimus induce autophagy at the tolerable dose and whether this dose induces synergism impact on chemotherapy or not [325,326].

In a study conducted by Wang et al. [327] a nanocarrier was used that could simultaneously overcome the double physiological barriers of tumors. The nanocarrier was designed as a pH-triggered reversible swelling-shrinking core and a matrix metallopeptidase 2 (MMP2) degradable shell for co-delivery of chemotherapeutics DOX and autophagy inhibitors (3-MA). This nanocarrier showed high penetration efficiency into the core of a tumor, both in vitro and in vivo. It also showed a synergistic anticancer property by combining DOX with 3-MA. 

In an experiment conducted by Rao et al., size-adjustable DSPE-PEG micelles containing DOX and wortmannin investigated for autophagy suppression and tumor inhibitory effects. Decreasing the level of LC3-II along with increasing the expression of p62 confirmed the successful decline of autophagy in B16F10 and 4T1 cell lines. Small particles maintain deep tissue penetration but rapid clearance from the bloodstream. By size increment, particles trapped in the extracellular matrix and prolong retention enhanced. This dual-drug nanoparticle demonstrated autophagy inhibition and anti-tumor activity in melanoma and breast cancer mouse models [8].

In other work by Lu et al. [328], synergistically therapeutic nanohybrids were constructed by combining amino-functionalized mesoporous silica nanoparticles (MSNs) with high loading capacity for a chemotherapeutic agent and gold NPs as pore blocker and ROS elicitor to eliminate human NSCLC. The nanohybrids were able to bring along the oxidative stress-induced autophagy with chemotherapy that provided a remarkable therapeutic approach.

In their study, AbdElhamid et al. [329] used a three multifunctional nanoplatform to develop a systemic co-delivery method for celecoxib (CXB) and rapamycin to induce growth inhibition of breast cancer cells. These therapeutic nanocapsules were prepared with an oil reservoir for dual loading of CXB and rapamycin, then electrostatically coated with oppositely charged polysaccharide (chondroitin) and protein (gelatin) coupled to fluorescent quantum dots. The fluorescent property of these nanostructures enabled tracing their internalization into cancer cells. Besides this, they showed significant toxicity against breast cancer cells.

In another survey, docetaxel as a supreme antitumor drug and LY294002 as an autophagy inhibitor loaded into PLGA NPs in order to resolve the low bioavailability and hydrophobicity of LY294002 and Docetaxel respectively. 155.3 nm PLGA (Docetaxel+LY294002) NPs accumulated in tumor site due to the EPR effect in mice bearing gastric cancer xenograft model. LY294002 inhibition of PI3K/ AKT signaling could suppress metastasis by downregulating MMP2, MMP9, and VEGF. This NP showed controlled release and high level of apoptosis in both xenograft and in situ gastric cancer mouse models [22].

Cisplatin is a potent medication for cancer, which has widely been used in different types of cancer. However, resistance to cisplatin is a major obstacle in tumor treatment procedure. Using autophagy inhibitors like wortmannin and siRNAs seems to be a promising strategy for reversing drug resistance and cellular sensitivity [7,26]. In a recent study, cisplatin and wortmannin were co-delivered within PLGA-PEG NPs. Significantly decrease of γH2AX foci as a DNA double-strand breaking marker attested wortmannin’s ability to inhibition of DNA repair and autophagy suppression. This dual drug reversed cisplatin resistance and improved chemoradiotherapy in PSOC and PROC murine models [26]. In another study, Lin et al. designed a prodrug aimed to resolve cisplatin drug resistance. DSPE-PEG nanoparticles loaded with cisplatin (Pt(IV)) and Beclin1 siRNA, and modified by DSPE-PEG and cRGD chain with an average diameter of 55 nm employed for efficient drug delivery and autophagy suppression in tumor cells. In the core of the particle, cisplatin conjugated with cationic peptide (KTGRKKRRQRRRG) covalently and then functionalized by bis(pyrene) and siRNA. This self-assembled prodrug with a mean diameter of 54 nm could powerfully silence Beclin1 both in vivo and in vitro. Decreased expression of Beclin1 along with a reduced level of LC3-II as well as a relative decline in ULK1 and TFEB1 mRNA levels resulted in enhanced chemotherapy, mitigated drug resistance, and tumor growth inhibition on the cisplatin-resistant tumor in xenograft mouse model [7]. Taking advantage of tumor tissues’ extraordinary energy demand could be a proper approach for targeting cancer cells. Tumor cells’ high metabolism leads to overexpressing albumin binding proteins for internalizing albumin as an amino rich source. Lu et al. prepared folic acid modified paclitaxel/CQ nanoparticles for improving transportation across blood-brain-barrier to target glioma cells. CQ effectively inhibited autophagy through endosomal acidification, increasing LC3-II to LC3-I level, interrupting over-expression of SQSTM1/P62, and autophagosome fusion with lysosome. Modified paclitaxel/CQ nanoparticles with 53 nm mean size induced apoptosis in glioblastoma multiforme LN229 and T98G mouse brain endothelial bEend.3 cells compared to paclitaxel alone [28].

Lately, Feng et al. developed PEGylated nanoliposomes carrying 5-fluorouracil as an anticancer drug and LY294002 an autophagy inhibitor for esophageal squamous cell carcinoma (ESCC) treatment. In this co-loaded cargo, LY294002 showed faster release than 5-fluorouracil. As a result, autophagy inhibition occurs prior to drug release, which leads to enhanced cancer cells sensitivity towards 5-fluorouracil. These nanoliposomes with an average size of 150 nm, extinguished the expression level of the anti-apoptotic protein Bcl-2 along with up-regulating the expression of caspase-3 and PARP on the esophageal cancer cell line, EC 9706. In vivo mice model indicated gradual release in the acidic environment of surrounding cancer cells and tumor growth delay compare to control group [27].

Taking everything into account, combination therapies with autophagy inducer and inhibitors need more investigation and their outcome in the clinic are, in part, a long way from pre-clinical investigations, due to the complicated condition of cancer in humans, compared with models of cancer in animals.

## 11. Nanocarriers as Autophagy Modulator: Challenging to Design Drug Delivery System

As mentioned earlier, liposomes have lipid nature, leading to the stimulation and formation of autophagic membranes which may be suitable to induce autophagy. However, it seems that they are not an appropriate candidate in autophagy inhibition. Gao et al. investigated the relationship between autophagy and cytotoxicity derived from polyethylenimine in MDCK (Madin-Darby canine kidney) and Chang liver cell lines [330]. They showed that inhibition of autophagy decreases the cytotoxicity induced by PEI, whereas autophagy induction enhances cell death, suggesting that autophagy is a key contributor for the enhancement of PEI cytotoxicity. Moreover, their results demonstrated that cytotoxicity derived from PEI-mediated autophagy acts through two steps: (1) early stage (3 h) with lysosome damage and (2) later stage (24 h) with mitochondrial damage. In another study, Li et al. investigated the effects of cationic PAMAM NPs on acute lung injury [331]. Their results demonstrated that cationic PAMAM NPs acts through Akt-TSC2-mTOR signaling pathway to induce autophagic cell death, resulting in the promotion of acute lung injury. It seems that autophagy inhibition is vital in generation of adverse effects of NPs. It is held that exposing to silica NPs is associated with development of pulmonary fibrosis. Investigation of molecular signaling pathways reveals that silica NPs are capable of inducing impairment in autophagy, resulting in apoptotic cell death in alveolar epithelial cells [332]. It appears that the aggregation of NPs after cell internalization negatively influences autophagy [333]. The modulatory effect of nanocarriers on autophagy can mediate their anti-inflammatory activity. Upconversion NPs encapsulating chlorin e6 effectively enhance the generation of ROS and subsequently, stimulate autophagy through PI3K/Akt/mTOR signaling pathway, resulting in down-regulation of pro-inflammatory factors (IL-12, TNF-α and iNOS) production [12]. Taking everything into account, NPs are able to regulate autophagy and this potential can be promisingly used in biomedical applications [334]. However, these nanoparticles could also trigger other organ toxicity tightly related with autophagy. For instance, ZnO NPs induce harmful impacts on gastrointestinal tract and autophagy stimulation is considered to be a potential candidate in ameliorating these toxic effects [335].

In brief, it is worth mentioning that nanoparticles are able to induce or inhibit autophagic cell fates [192,336]. Therefore, autophagy nano-inhibitors should not be used for delivery of autophagy inducers and autophagy nano-inducers are not good options as nanocarriers for autophagy inhibitors. 

## 12. Conclusions and Remarks

Nowadays, autophagy is the focus of attention owing to its homeostasis role in cells. Autophagy not only guarantees cell survival through the engulfment of damaged organelles and proteins and, thereby, its cargo biodegradation, but it also acts as a cell-assure valve, which leads to saving cellular power supplies. Considering the critical importance of the equilibrium of autophagy in cell, its disturbance leads to a great number of diseases and disorders. Therefore, an accurate understanding of the real causes of the diseases is important. Meantime, scientists are attempting to discover the underlying cause of the disease and find the effective remedies for them. However, the great majority of these molecules have poor bioavailability, mainly associated with their low solubility in water. However, it seems that nanotechnology may overcome these obstacles. 

Autophagy has diverse check points, and medications can act through them to reverse the autophagy function. For example, it can modulate through the mTOR, AMPK, class III PI3K and MAPK signaling pathways. There are some conventional drugs to influence autophagy as an inducer or inhibitor including metformin, simvastatin, everolimus, temsirolimus, dactolizib, CQ etc. It is worth mentioning that there is controversy on the mechanism of autophagy in cancer progression and it seems that it is stage-dependent. In order to prove the finding, reference may be made to the protective potential of metformin in cancer, while it is not effective when the cancer is triggered and progressed. This review presets several pharmaceutics involved in the treatment of cancer by emphasis on their autophagy modulatory roles, and also introduces some drug nanocarriers to improve the effectiveness of conventional pharmaceutics and overcome to their obstacles. Notably, encapsulation of conventional autophagy modulators into the nanocarriers leads to design of a theranostic-controlled release nanoplatform that targets damaged cells and selectively responds to stimuli with high efficacy. These reports delineate the promise of nanocarriers and combination therapy as a novel strategy to treat cancer and other pathological conditions through autophagy modulation.

## Figures and Tables

**Table 1:** Nanocarriers for autophagy modulators delivery.

Autophagy Inducers/Inhibitors	Nano/Micro-Carriers	Co-Delivered Drug/siRNA	Disease	Targeting Agent	In Vitro/In Vivo	Major Outcomes	Refs
Rapamycin	Zain-lactoferrin micelles	Wogonin	Breast cancer	-	In vitro (MCF-7 breast cancer cells) and in vivo (Ehrlich ascites tumor animal model)	Inhibition of tumor growth with minimized side effects	[230]
Rapamycin	Hollow Fe₃O₄/Graphene Oxide Nanocomposites	-	-	-	In vitro (HepG2 cells)	High EE (84.92%), good stability and great cytotoxicity against HepG2 cells	[231]
Rapamycin	Berunda Polypeptides	-	Breast Cancer	-	In vivo (human MDA-MB-468 orthotopic breast cancer xenografts) and in vitro	Suppression of tumor growth and decreased viability of tumor cells	[232]
Rapamycin	Elastin-like Polypeptide NPs	Integrins	Breast cancer	-	In vivo (MDA-MB-468 breast tumor)	Inhibition of tumor growth in a higher level (3 folds)	[233]
Rapamycin	Immunoliposomes	-	Inflammatory disorder	anti-E-selectin	In vitro (activated endothelial cells)	Inhibition of endothelial cells migration and proliferation as well as inflammatory cytokines expression	[234]
Rapamycin	Polymer-lipid hybrid NPs	-	Hemangioma	-	In vitro (human hemangioma endothelial cells) and in vivo (female Balb/c mice)	Effective binding with HemECs and remarkable proliferation inhibition and decreased expression of angiogenic factors as well as decreased hemangioma volume, weight and microvessel density in in vivo	[235]
Rapamycin	Graphene oxides wrapped with PEGylated lipid bilayer	DOX	-	-	In vitro (MCF-7, MDA-MB-221 and BT474 cells)	Treatment and prevention of resistant cancer cells by up-regulating Bax, P21, P53, and caspase-3 and apoptosis induction	[236]
Rapamycin	Liposomes	Polypyrrol	Breast cancer	trastuzumab	In vitro (BT-474 cells)	Overcoming against drug resistance in breast cancer and higher therapeutic efficacy in breast cancer cells	[237]
Rapamycin	Lactose-wrapped calcium carbonate NPs	-	Cellular senescence	CD9	In vitro (senescent cells)	Prevention of cellular senescence and improved proliferation of aged cells	[238]
Rapamycin	PEG-PCL NPs	-	Pulmonary arterial hypertension (PAH)	-	In vivo (rat model of PAH)	High accumulation of nanoparticles in lung, attenuation PAH development and also decreased systemic side effects compared to the free rapamycin	[239]
Rapamycin	Immunoliposomes	Paclitaxel	Breast cancer	Anti-HER2	In vitro (HER2(+) breast cancer cells and triple negative cancer cells) and in vivo (nude mice with HER2(+) breast cancer cells)	High cytotoxicity due to enhanced uptake through HER2 binding and decreased tumor volume in vivo	[240]
Rapamycin	PLGA-PCL NPs	-	Breast cancer	-	In vitro MCF-7 and human lymphocyte cell (Jurkat cells)	Inhibition of cell proliferation in MCF-7 cells, suppression of cell growth in Jurkat cells and simultaneously, maintaining the bioactivity of rapamycin	[241]
Rapamycin	PLGA NPs	-	Venous neointimal hyperplasia	Pericardial patches	In vitro (human smooth muscle cells) and in vivo (male Wistar rats)	Sustained rapamycin delivery and subsequently, less neointimal hyperplasia, less smooth muscle cells proliferation and lower infiltrating cells and simultaneously, maintaining endothelization	[242]
Rapamycin	PEGylated liposomes	-	Cellular senescence	CD9 monoclonal antibody	In vitro (CD9 receptor-overexpressing cells)	Promotion of cell proliferation and reduction in the number of cells that express the senescence-associated-galactosidase, showing higher anti-senescence activity of CD9-targeted liposome compared to the free rapamycin and conventional liposomes	[243]
Rapamycin	PEO/PDLLA nanofibers	-	Glioblastoma	-	In vitro (U251 and U87 human glioblastoma cell lines)	Local sustained delivery of rapamycin, showing potential targeted delivery systems for glioblastoma treatment	[244]
Rapamycin	Polymeric NPs	Piperine	Breast cancer	-	In vitro (breast cancer cells)	Increased cellular uptake and bioavailability of rapamycin as well as decreased viability of cancer cells	[245]
Rapamycin	Lipid-polyaniline NPs	1,1-dioctadecyl-3,3,3,3-tetramethylindotricarbocyanine iodide (DiR)	Cancer	-	In vitro (HeLa cells) and in vivo (HeLa tumor bearing mice)	High antiangiogenic effect and great cytotoxicity as well as decreased tumor growth	[246]
Rapamycin	Polymeric Micelle	Paclitaxel and 17-allylamino-17-demethoxygeldaramycin (17-AAG)	Cancer	-	In vitro (A549 cells)	Inhibition of A549 tumor growth, increased cytotoxicity and enhanced radiosensitizing effect	[247]
Rapamycin	poly(ethylene glycol)-shelled NPs	-	Aortic Aneurysm	-	In vivo (experimental aortic aneurysm in rat)	Remarkable inhibition of activities of matrix metalloproteinase and expression of inflammatory cytokines, showing their potential in targeting aortic aneurysm	[248]
Rapamycin	Acetalated b-CD (Ac-bCDs)-based NPs	-	Atherosclerosis	-	In vitro (smooth muscle cells) and in vivo (apoliproprotein E-deficient (ApoE) mice)	Decreased formation of atherosclerotic lesions, increased stability of plaques, decreased level of pro-inflammatory factors and suppression of mTORC1	[249]
Rapamycin	Immunoliposomes	-	Breast cancer	Trastuzumab	In vitro (triple negative MDA-MB-231 and SKBR3cell lines)	High cytotoxicity against breast cancer cells	[250]
Rapamycin	Thermal sensitive liposomes	Indocyanine Green	-	-	In vitro (HeLa and HUVEC cells) and in vivo (HeLa cell bearing mice)	Great drug accumulation and cytotoxicity in vitro experiment and inhibited tumor growth in vivo with minimal side effects	[251]
Rapamycin	PLGA particles	Isoniazid and rifabutin	-	-	In vivo (infected mice) and in vitro (THP-1 human monocytes)	Stimulating more autophagy in infected macrophages, decreased bacterial burden in lung and spleen and inducing phagosome-lysosome fusion	[252]
Rapamycin	Liposomes	Paclitaxel	Breast cancer	-	In vitro (cancer 4T1 breast cancer cell line) and in vivo (4T1-tumor bearing mice)	Higher cytotoxicity against 4T1 cells and decreased tumor growth and viability in mice	[253]
Rapamycin	Human serum albumin NPs	Split luciferase reporter			Combined image guided monitoring the pharmacokinetics		[254]
Rapamycin	Solid Lipid NPs	-	-	-	In vitro (SH-SY5Y neuroblastoma cells)	High cellular uptake, sustained release and higher mTORC1 inhibition	[255]
Rapamycin	Biodegradable intraocular device	-	-	-	In vivo (New Zeland white rabbits)	Prolonged release, good stability and good ocular compatibility	[256]
Rapamycin	Lipid SAINT-O-Somes	-	-	Anti-VCAM-1	In vitro (ABN12 and MPC-5 cell lines)	Remarkable inhibition of AB8/cell migration in targeted nanocarriers	[257]
Rapamycin	Nanoemulsions	-	-	-	In vitro (SKBR3 and Caco-2 cell lines)	Great cytotoxicity again SKBR-3 cell and good uptake by Caco-2 cells	[258]
Rapamycin	Microsphere	-	Kidney disease	-	In vivo (rat model of renal ischemic/reperfusion injury)	Lack of adverse effects, decreased macrophage infiltration and lower amount of myofibroblasts in kidney	[259]
Rapamycin	magnetic Fe3O4/carboxymethylchitosan NPs	-	Cancer	-	In vitro (liver cell line LO_2_ and human hepatocarcinoma cell line HepG2)	Sustained release of rapamycin, higher cytotoxicity against LO_2_ and HepG_2_ cells, increased cellular uptake and decreased damage to normal cells	[260]
Rapamycin	Nanoliposomal	CPT-11	Brain tumor	-	In vivo (rodent orthotopic brain tumor xenografts)	Significant efficacy in increasing survival with minimal side effects	[261]
Rapamycin	Liposome	-	-	-	In vitro	High rapamycin encapsulation rate, good reproducibility and sustained release	[262]
Rapamycin	Aerosol treatment	3-bromopyruvate	Lung cancer	-	In vitro (human non-small cell lung cancer (NSCLC)) and in vivo (mice with lung cancer0	Remarkable inhibition of cell proliferation, decreased glycolytic activity, resulting in antitumor effect	[263]
Rapamycin	Porous silicon microparticles	-	-	-	In vivo (rabbit)	Great rapamycin loading, increased bioavailability and simultaneously, maintaining clear optical media and normal histology of retina	[264]
Rapamycin	Subcapsular microspheres	-	Chronic kidney disease	-	In vivo (ureter-obstructed rats)	Decreased intrarenal mTOR activity, over-expression of fibrotic genes, myofibroblast accumulation and T-lymphocyte infiltration and subsequently, successful inhibition of local fibrotic response	[265]
Everolimus	Chitosan NPs	-	Bronchiolitis obliterans syndrome (BOS)	-	In vitro (CD44-overexpressing mesenchymal cells)	Great properties in terms of average size (≤200 nm), good zeta potential (-30.9mV) and sustained release behavior as well as good uptake by mesenchymal cells	[266]
Everolimus	Polymeric NPs	Paclitaxel	Breast cancer	-	In vitro (MCF-7 and SKBR3 cells)	Synergistic effect on inhibiting the growth and decreasing viability of breast cancer cells	[267]
Everolimus	mPEGhexPLA nanocarriers	-	Autoimmune uveoretinitis (EAU)	-	In vivo (B10,RIII mice)	Significant decrease in EAU severity in both eyes and decreased secretion of IL-10 and CD4^+^ CD25^+^ FoxP3^+^	[268]
3-methyladenine	Metal-organic framework NPs	-	Cancer	-	In vitro (HeLa cells) and in vivo (nude mice)	Significant inhibition of autophagosome formation in HeLa cells and higher anti-tumor activity and inhibition of Beclin-1 and LC3 in mice	[269]
Cholorquine diphosphate	Metal-organic framework NPs	-	Cancer	Methoxy poly (ethylene glycol)-folate (FA-PEG)	In vitro (HeLa cells)	Remarkable inhibition of autophagosome formation and autophagy flux	[270]
Dactolisib	PLGA-PEG NPs	-	-	Anti-human E-selectin antibody	In vitro (TNF-activated endothelial cells)	High cellular uptake and great anti-inflammatory effects	[271]
